# Thermogenesis is limited by cellular competence

**DOI:** 10.3389/fcell.2026.1784579

**Published:** 2026-02-20

**Authors:** Aaron C. Brown

**Affiliations:** 1 Center for Molecular Medicine, MaineHealth Institute for Research, Scarborough, ME, United States; 2 Graduate School of Biomedical Sciences and Engineering, The University of Maine, Orono, ME, United States; 3 Tufts University, School of Medicine, Boston, MA, United States

**Keywords:** aging, beige adipocytes, cellular competence, mitochondrial quality control, obesity, thermogenesis

## Abstract

Beige adipocytes have emerged as an attractive therapeutic target for metabolic disease due to their inducible thermogenic capacity and developmental plasticity. However, despite substantial advances in understanding the molecular pathways that activate thermogenesis, most thermogenic strategies have shown limited durability in pathological settings. This article integrates recent discoveries in adipocyte cell biology to argue that thermogenic failure reflects a loss of cellular competence rather than insufficient stimulation. We review emerging evidence demonstrating that mitochondrial capacity, intracellular signaling fidelity, and vesicle trafficking impose critical cell-intrinsic constraints on beige adipocyte function, particularly in obesity and aging. These insights highlight why chronic, systemic activation strategies often fail to produce sustained metabolic benefits. Drawing on principles from developmental biology, we propose that restoring thermogenic function will require precision control of adipocyte cell state, including spatially and temporally defined modulation of signaling pathways. Emerging technologies enabling reversible, cell-targeted control of adipocyte function, coupled with human cell-based models, offer new opportunities to overcome current limitations. Together, this perspective emphasizes that beige adipocytes are not merely thermogenic effectors, but dynamic cellular systems whose therapeutic potential depends on maintaining or restoring adaptive plasticity.

## Introduction

Obesity and type 2 diabetes arise from chronic imbalance between energy intake and expenditure and are characterized by impaired metabolic flexibility. Thermogenic adipocytes provide a physiological mechanism to increase energy expenditure and counter this imbalance. These cells comprise two related but distinct populations: classical brown adipocytes, which are developmentally specified and reside in dedicated depots, and inducible beige adipocytes, which emerge within white adipose tissue in response to environmental or hormonal cues (Kajimura et al., 2015; Wu et al., 2012). Both cell types dissipate energy as heat, and their activation is linked to improved systemic glucose and lipid metabolism (Cannon and Nedergaard, 2004; Sidossis and Kajimura, 2015). In humans, functional imaging and physiological studies demonstrate that thermogenic adipose depots can be recruited by cold exposure and pharmacological stimulation (Cypess et al., 2009; van Marken Lichtenbelt et al., 2009; Cypess et al., 2015), and their activation produces measurable systemic metabolic effects (Chondronikola et al., 2016). In contrast to laboratory mice, which are typically housed below thermoneutrality and therefore experience persistent cold stress, thermogenic adipose activation in humans is generally inducible rather than continuously engaged, helping explain why responses observed in preclinical models are often stronger and more durable than in humans.

Across studies, most readily detectable thermogenic activity in humans localizes to classical brown adipose depots, while the extent to which beige adipocytes contribute to thermogenesis within subcutaneous white adipose tissue, the depot most commonly associated with beige recruitment, remains incompletely defined under typical physiological conditions (Cypess et al., 2009; van Marken Lichtenbelt et al., 2009; Cypess et al., 2015). Nonetheless, molecular and transcriptomic analyses of human thermogenic depots reveal gene-expression signatures resembling murine beige adipocytes, suggesting partial conservation of inducible thermogenic programs (Wu et al., 2012; Sidossis and Kajimura, 2015; Sharp et al., 2012; Lee et al., 2014; Shinoda et al., 2015). Human subcutaneous adipose-derived precursor cells can be induced to adopt thermogenic beige-like phenotypes *in vitro*, demonstrating retained cell-autonomous browning potential (Lee et al., 2014; Anatildes da Silva de Paula et al., 2025). Pathological browning of white adipose tissue has been documented in humans across multiple chronic disease contexts, including severe burn injury, cancers, kidney disease, SARS-CoV-2 infection, and sepsis, indicating that human white adipose tissue retains the capacity for thermogenic remodeling *in vivo* (Zhang et al., 2026). However, these responses often occur under extreme systemic stress, highlighting the challenge of restoring thermogenic sensitivity and durability under physiologically relevant or therapeutically tolerable stimulation.

**FIGURE 1:**
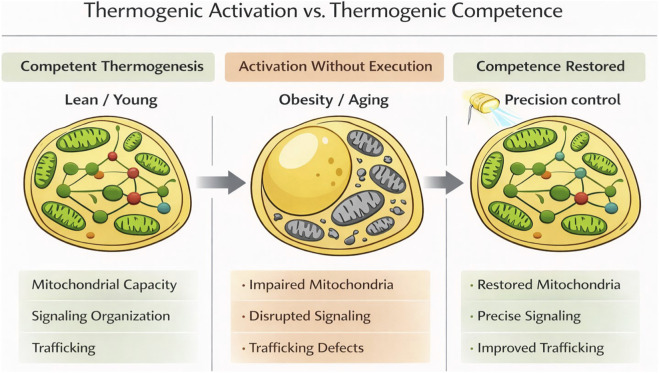
Thermogenic competence is governed by adipocyte cellular state. Conceptual model illustrating the distinction between thermogenic activation and thermogenic competence across metabolic states. Left, in lean or young adipocytes, intact intracellular organization, preserved mitochondrial capacity, and coordinated signaling and trafficking enable effective execution of thermogenic programs, such that acute stimulation results in robust heat production. Middle, in obesity and aging, mitochondrial dysfunction, disrupted signaling organization, and trafficking defects constrain thermogenic execution, leading to “activation without execution” despite the presence of upstream thermogenic cues. Right, precision control of adipocyte cell state through spatially and temporally defined, reversible perturbations (for example, optogenetic approaches) enables targeted interrogation and restoration of specific components of mitochondrial function, signaling organization, and trafficking capacity, enabling recovery of thermogenic competence. The model emphasizes that thermogenic failure in metabolic disease reflects loss of cellular adaptability rather than absence of thermogenic pathways, and that durable therapeutic benefit requires restoring cellular competence rather than sustained pathway activation.

Human imaging and physiological studies show that the magnitude and durability of thermogenic responses are reduced in obesity and aging, with diminished cold-induced glucose uptake and blunted metabolic responses in obese individuals and older adults (Sidossis and Kajimura, 2015; Valentine et al., 2022; Alcala et al., 2019; Yoneshiro et al., 2011; Orava et al., 2013). Interpretation of human imaging studies is shaped by technical limitations, as PET detection is optimized for dense, highly oxidative depots and may underreport more spatially diffuse or lower-density thermogenic remodeling within white adipose tissue, particularly in subcutaneous depots. These considerations highlight the need to complement imaging with cell- and tissue-level readouts that determine whether adipocytes are capable of executing thermogenesis even when whole-depot signals appear modest. Taken together, important gaps remain in our understanding of thermogenic remodeling in humans. From a therapeutic perspective, the anatomical accessibility and plasticity of subcutaneous adipose tissue suggest that white adipose depots remain a practical target for strategies aimed at restoring thermogenic competence through localized or cell-targeted intervention, particularly if cellular competence can be restored locally (Sidossis and Kajimura, 2015). These observations suggest that limitations in thermogenic adipocytes extend beyond pathway activation alone and instead reflect fundamental constraints imposed by cellular state and intracellular capacity. This distinction motivates a shift from viewing thermogenesis as a problem of pathway activation to one of cellular competence. These concepts are summarized in Figure 1, which presents a conceptual framework linking adipocyte cellular state to thermogenic execution in lean, obese, and precision-controlled contexts.

In this perspective, cellular competence is defined as the measurable capacity of adipocytes to execute and sustain thermogenesis given intact upstream stimulation. This competence reflects the state of intracellular systems, including mitochondrial architecture, signaling organization, vesicle trafficking, and stress management, which together determine whether thermogenic programs can be functionally realized and maintained (Table 1). Throughout this Perspective, we use “activation” to refer to acute engagement of thermogenic signaling pathways, “maintenance” to describe sustained preservation of the thermogenic cell state, and “regression” or “whitening” to denote loss of thermogenic features following withdrawal of activating cues.

**TABLE 1:** Operational dimensions of thermogenic cellular competence.

Dimension	Operational features	Representative readouts
Mitochondrial respiratory capacity	Ability to support sustained oxidative flux	Respiratory reserve capacity; electron transport chain activity; mitochondrial membrane potential
Mitochondrial architecture	Structural organization enabling efficient respiration	Cristae density and organization; fusion-fission balance; mitochondrial network morphology
Mitochondrial turnover	Balance between mitochondrial renewal and clearance	Mitophagy–biogenesis flux; Parkin recruitment; LC3-mitochondria colocalization; mitochondrial DNA content
Signaling compartmentation	Spatial organization of thermogenic signaling	cAMP microdomain amplitude and duration; phosphodiesterase localization; compartment-specific PKA activity
Receptor trafficking dynamics	Capacity to sustain productive signaling	β-adrenergic receptor internalization, recycling, and resensitization kinetics
Vesicle trafficking capacity	Intracellular organization and stress adaptation	Endosomal sorting efficiency; multivesicular body formation; Rab-dependent vesicle docking and release
Cellular stress tolerance	Ability to remodel under metabolic load	Endoplasmic reticulum stress markers; proteostasis capacity; oxidative stress responses

cellular competence reflects the capacity of adipocytes to execute and sustain thermogenesis given intact upstream stimulation. The listed dimensions and representative readouts illustrate experimentally tractable features that together define the intracellular ceiling on thermogenic execution, distinct from transcriptional activation of thermogenic pathways.

## What recent cell biology has revealed about inducible thermogenesis

Recent advances in adipocyte cell biology have shifted emphasis from whether thermogenic programs can be initiated to whether they can be executed and maintained under metabolic stress, revealing thermogenesis to be an organelle- and systems-level phenotype constrained by intracellular capacity and coordination (Kajimura et al., 2015; Liesa and Shirihai, 2013; De Jong et al., 2025; Aquilano et al., 2023; Bond et al., 2022). While much of the molecular machinery underlying thermogenesis has been defined using classical brown adipocytes, inducible beige adipocytes provide a particularly informative experimental context in which to interrogate thermogenic failure, owing to their reversible thermogenic state in preclinical models. In this framework, β-adrenergic inputs may remain intact and transcriptionally productive, yet sustained heat production depends on whether adipocytes can preserve the intracellular organization that couples signaling to substrate mobilization and mitochondrial respiration (Anatildes da Silva de Paula et al., 2025; Song et al., 2023). The strength of evidence across these mechanisms varies, ranging from direct genetic perturbation to associative and hypothesis-driven observations.

## Mitochondrial competence as a limiting factor

Thermogenesis is ultimately executed at the level of mitochondrial structure and function. Early work established that mitochondrial dynamics are fundamental regulators of cellular energy expenditure (Liesa and Shirihai, 2013), but more recent studies have identified specific molecular nodes that directly constrain thermogenic output. In particular, the balance between mitochondrial fusion and fission has emerged as a critical determinant of adipocyte metabolic flexibility. Genetic disruption of inner mitochondrial membrane organization through loss of OPA1, a key mediator of mitochondrial inner membrane fusion and cristae maintenance, in thermogenic adipose tissue leads to altered cristae ultrastructure, impaired mitochondrial respiration, and reduced thermogenic capacity, despite preserved upstream activation signals (Pereira et al., 2021; Bean et al., 2021). Importantly, mitochondrial quality control is not merely permissive but required for sustained thermogenic activation, as mitophagy is induced in brown adipose tissue during cold challenge and is essential for maintaining mitochondrial homeostasis and respiratory competence under prolonged metabolic demand (Lu Y. et al., 2018). Conversely, excessive mitochondrial fragmentation driven by DRP1, the principal regulator of mitochondrial fission, suppresses substrate oxidation and thermogenic efficiency in adipose tissue under obesogenic conditions (Xia et al., 2024). In beige adipocytes, mitochondrial turnover plays an even more prominent role, as Parkin-dependent mitophagy actively regulates thermogenic cell-state maintenance through a mechanism independent of canonical thermogenic effectors, with enhanced mitochondrial clearance contributing to thermogenic regression (Lu X. et al., 2018). Together, these findings indicate that mitochondrial network architecture causally sets an upper bound on thermogenic flux that limits execution even when thermogenic pathways are engaged.

## Beige adipocytes as a sensitive readout of mitochondrial quality control

In experimental systems, beige adipocytes are particularly sensitive to perturbations in mitochondrial homeostasis because their thermogenic identity is tightly coupled to mitochondrial abundance. A landmark study demonstrated that beige adipocyte maintenance is actively regulated by autophagy-driven mitochondrial clearance, with enhanced mitophagy triggering regression to a white-like state following withdrawal of thermogenic stimuli (Altshuler-Keylin et al., 2016). This work provided a direct mechanistic explanation for the reversibility of beige adipocyte thermogenesis and established mitophagy as a *bona fide* regulator of adipocyte cell state. Collectively, these findings position beige adipocytes as a highly sensitive indicator of mitochondrial quality-control capacity within adipose tissue.

## Intracellular signaling fidelity, compartmentation, and trafficking

Evidence linking compartmentalized cAMP signaling to thermogenic execution is largely associative and inferential, derived from live-cell imaging, pharmacological perturbation, and correlative remodeling of signaling microdomains. A second major constraint on thermogenic execution is the fidelity of intracellular signaling. β-adrenergic signaling in adipocytes relies on precise spatial and temporal control of cAMP production and downstream effector activation, rather than uniform cytosolic signaling. Early work established the complexity and adaptability of β-adrenergic signaling networks in adipocytes (Collins, 2022), and more recent syntheses now emphasize compartmentalized β-adrenergic/cAMP signaling as a central organizing principle linking receptor activation to metabolic outputs across white and brown adipocytes (De Jong et al., 2025). Building on this conceptual framework, recent experimental advances have provided mechanistic insight into how intracellular signal organization shapes the execution of thermogenesis.

Live-cell imaging studies have demonstrated that cAMP signaling in adipocytes is organized into discrete microdomains shaped by phosphodiesterase activity and subcellular localization, including endosomal compartments (De Jong et al., 2023). Importantly, differentiation into thermogenic adipocytes is accompanied by remodeling of these cAMP microdomains, such that identical receptor inputs are translated into distinct metabolic outputs depending on intracellular signal routing (Kannabiran et al., 2020). This provides a mechanistically informed explanation for why chronic β-adrenergic stimulation can lose effectiveness even when receptors remain present. Signaling execution may fail not because receptors are absent, but because intracellular signal organization may become maladaptive.

Endosomal trafficking plays a central role in shaping this organization. β-adrenergic receptors undergo regulated internalization and recycling, and endocytic sorting can reshape the magnitude, duration, and spatial origin of downstream signaling, as established for GPCRs more broadly (Sorkin and von Zastrow, 2009). In the context of obesity and aging, reduced β-adrenergic responsiveness in adipose tissue provides a physiological setting in which defects in intracellular trafficking and signaling compartmentation further erode durable thermogenic outputs (Collins, 2022; Large et al., 1999; Lonnqvist et al., 1990). Thus, signaling failure likely reflects not only receptor desensitization but broader breakdowns in intracellular organization required to sustain thermogenic execution.

## Vesicle trafficking and extracellular vesicles as adaptive regulators

Vesicle trafficking and extracellular vesicle (EV) secretion are increasingly recognized as being associated with metabolic adaptation. Classical cell biology has established that exosome biogenesis is a regulated, Rab-dependent process essential for cellular homeostasis under stress (Colombo et al., 2014), with Rab27a and Rab27b identified as key regulators of multivesicular body docking and fusion (Ostrows et al., 2010). Within adipose biology, recent high-profile studies have expanded the role of EVs from biomarkers to active regulators of thermogenic physiology. In this context, extracellular vesicles should be distinguished as either biomarkers or readouts of thermogenic activation and cellular stress, or as functional mediators of intercellular communication, with substantially stronger evidence supporting the former than the latter in most settings. Brown adipose tissue releases EVs whose protein cargo reflects thermogenic activity and mitochondrial status (Camino et al., 2022), and in humans, activation of thermogenic adipocytes is associated with increased release of EVs containing mitochondrial proteins, supporting the use of EVs as indicators of intracellular metabolic remodeling rather than definitive proof of downstream effector function (Leow et al., 2022).

Importantly, extracellular vesicle pathways intersect with intracellular competence because the ability of adipocytes to produce and secrete EVs depends on intact endosomal sorting, membrane trafficking, and lipid handling (Colombo et al., 2014). These same processes are required to sustain thermogenic remodeling and maintain cellular homeostasis under high metabolic demand. EV release by thermogenic adipocytes is therefore best interpreted as both a readout and a component of preserved intracellular organization, rather than definitive evidence that extracellular vesicles act as primary endocrine drivers of thermogenic output. Consistent with this view, cold-activated brown adipose tissue releases EVs that deliver regulatory microRNAs to the liver, suggesting a role for EVs in coordinating systemic metabolic responses, rather than serving as primary determinants of thermogenic capacity (Xu et al., 2023).

Importantly, EV abundance and cargo composition are influenced by inflammatory state, cellular stress, clearance kinetics, and depot- or cell-of-origin heterogeneity, complicating interpretation of EVs as direct functional effectors of thermogenic adaptation (Bond et al., 2022). In obesity, impairment of vesicle trafficking may therefore compromise both cell-intrinsic homeostasis and adaptive interorgan communication.

## Convergence of obesity and aging on intracellular capacity

A unifying theme emerging from these studies is convergence. Obesity and aging simultaneously erode the cellular infrastructure required for sustained thermogenic remodeling, including mitochondrial homeostasis, signaling organization, and trafficking capacity (Hepler and Gupta, 2017; Ou et al., 2022). In obesity, diminished adrenergic responsiveness and catecholamine resistance provide a clear physiological context in which upstream cues may be present yet downstream execution becomes limited (Valentine et al., 2022; Alcala et al., 2019). In aging, thermogenic adipose activity declines in humans and is further constrained by age-associated changes in the tissue microenvironment that compromise brown adipose function (Yoneshiro et al., 2011; Feng et al., 2023). As a result, upstream activation pathways may remain inducible, yet thermogenic execution becomes fragile and transient. In humans, cold-induced activation of thermogenic adipose tissue can be detected across populations, but both obesity and aging are associated with reduced magnitude and altered metabolic consequences of this activation (Yoneshiro et al., 2011; Orava et al., 2013). This convergence provides a mechanistic framework for why thermogenic responses decline in metabolic disease even when canonical signaling pathways appear intact.

## Non-cell-autonomous constraints on thermogenic execution *in vivo*

Non-cell-autonomous constraints impose important limits on thermogenic execution *in vivo*, as thermogenic adipocytes respond to and participate in systemic neural, vascular, immune, and endocrine signaling networks (Kajimura et al., 2015; Villarroya et al., 2017). These include sympathetic innervation, vascularization and oxygen delivery, immune cell composition and extracellular matrix remodeling, endocrine and central nervous system regulation, and the availability of metabolic substrates (Bartness et al., 2014; Sun et al., 2012; Trayhurn, 2013). In obesity and aging, reductions in sympathetic tone, impaired vascular remodeling and hypoxia, chronic inflammation, and fibrosis can blunt thermogenic responses, helping explain why systemic activation strategies often produce modest or transient effects (Yoneshiro et al., 2011; Orava et al., 2013). At the same time, the existence of these constraints does not preclude restoration of thermogenic capacity at the cellular level. When signaling can be engaged with sufficient spatial and temporal precision, thermogenic programs can, in principle, be activated within adipocytes even under suboptimal systemic conditions (Tajima et al., 2020; Doucette et al., 2023). In this framework, cellular competence remains a necessary foundation for thermogenesis and a tractable target for precision interventions that act locally within adipose tissue, rather than relying on intact global regulation.

## Why most thermogenic therapies fail

Most thermogenic therapies have been developed under the implicit assumption that activating upstream thermogenic signaling pathways is sufficient to restore energy expenditure. In young, lean preclinical models, acute activation strategies such as cold exposure, β-adrenergic agonism, or genetic sensitization robustly induce thermogenic programs and increase energy expenditure (Cannon and Nedergaard, 2004; Bachman et al., 2002). However, in pathological settings, particularly obesity and aging in humans, “failure” of thermogenic therapies has most commonly reflected limited magnitude of energy expenditure increases, poor durability with chronic stimulation, and dose-limiting safety or tolerability concerns, rather than failure to activate thermogenic pathways *per se*.

Translation to humans and metabolically diseased states has revealed fundamental limitations of this approach. In humans, pharmacological activation of β3-adrenergic signaling can activate thermogenic adipose tissue but produces modest or transient increases in energy expenditure and is frequently accompanied by cardiovascular side effects, limiting both efficacy and durability (Sidossis and Kajimura, 2015; Cypess et al., 2015). These outcomes are consistent with the interpretation that chronic stimulation targets adipocytes whose intracellular execution machinery is already compromised by obesity and aging, thereby contributing to catecholamine resistance and adaptive dampening despite preserved receptor expression and ligand availability (Collins, 2022; Large et al., 1999; Lonnqvist et al., 1990).

Experimental design further amplifies this disconnect. Many preclinical studies rely on cold exposure, short-term pharmacological treatments, or genetically sensitized models that bypass pathological constraints on cellular capacity. While valuable for defining thermogenic pathways, these approaches systematically overestimate therapeutic durability by masking the loss of mitochondrial competence, signaling fidelity, and adipocyte remodeling capacity that characterizes metabolic disease (Alcala et al., 2019; Hepler and Gupta, 2017). In contrast, studies in humans demonstrate that thermogenic adipocyte recruitment and metabolic responsiveness are blunted and less persistent in obese individuals, even when upstream thermogenic cues remain inducible (Orava et al., 2013).

Taken together, these observations indicate that thermogenic failure reflects not the absence of thermogenic pathways, but the inability of diseased adipocytes to execute and sustain thermogenesis under chronic stimulation (Sidossis and Kajimura, 2015; Collins, 2022). In contrast to systemic strategies that rely on sustained pathway activation, precision control approaches seek to restore thermogenic function by aligning signal timing, duration, and context with the adaptive capacity of adipocytes compromised by obesity and aging.

## Precision control of cell state as a solution

An alternative framework is to view thermogenesis as a problem of cell state rather than pathway activation. Developmental biology has long emphasized that cell fate and functional competence are governed by the timing, duration, amplitude, and contextual integration of signaling inputs, rather than signal presence alone (Sagner and Briscoe, 2017). More recent work has further refined this view by demonstrating that dynamic signaling patterns and temporal integration of pathway activity actively shape cell behaviors and fate decisions during development (Sonnen and Janda, 2021), with direct experimental evidence showing that signaling history itself can determine downstream cell-state outcomes (Teague et al., 2023). Applied to adipocytes, these principles suggest that restoring thermogenic capacity in obesity and aging may require reestablishing intracellular conditions that permit adaptive remodeling, rather than simply reactivating canonical pathways.

Precision control over signaling, both spatially and temporally, therefore represents a potential strategy to align thermogenic activation with the diminished adaptive capacity of adipocytes in metabolic disease. Temporally defined and reversible activation may allow mitochondrial architecture, vesicle trafficking pathways, and transcriptional programs to adapt progressively without triggering the maladaptive stress responses associated with chronic stimulation. In this view, therapeutic success shifts from forcing thermogenic output to restoring the cellular competence required to sustain it. These concepts yield specific, experimentally testable predictions (Box 1).

BOX 1 **Testable predictions for precision control of thermogenic competence.**

1. Pulsatile or temporally defined stimulation will better preserve cellular competence than sustained stimulation, as assessed by mitochondrial respiratory reserve, cristae integrity, and fusion-fission balance following repeated activation cycles.
2. Restoration of competence will be reflected by improved signaling fidelity rather than increased pathway amplitude, including stabilized cAMP microdomain organization and preserved receptor trafficking kinetics with reduced desensitization.
3. If cellular competence is limiting, improvements in competence readouts will predict more durable thermogenic execution under matched stimulation conditions, dissociating execution from upstream ligand availability.

Key assays include mitochondrial stress tests or microcalorimetry, quantitative imaging of mitochondrial ultrastructure and dynamics, live-cell cAMP reporters, receptor internalization and recycling assays, and complementary stress or proteostasis markers in human iPSC-derived beige adipocytes and disease-relevant mouse models.

Against this backdrop, while beige adipocytes provide a uniquely tractable system for studying reversible thermogenic competence, validation in classical brown adipocyte models will be essential to determine which mechanisms generalize across thermogenic adipocyte lineages. Human cell-based systems provide a critical platform for testing this concept. Human pluripotent stem cell-derived adipocyte systems enable scalable generation of white and thermogenic adipocytes and provide experimentally tractable models for interrogating how controlled perturbations are executed in human cells (Ahfeldt et al., 2012). More recently, iPSC-derived beige adipocyte models have extended this approach by providing a renewable and expandable source of human thermogenic adipocytes, enabling systematic manipulation of signal timing, duration, and amplitude under conditions that are difficult to achieve *in vivo* (Guenantin et al., 2017; Su et al., 2018). Although cellular reprogramming resets aspects of age-associated dysfunction (Su et al., 2018), these systems serve as a reference state against which loss of adaptive capacity in obese or aged adipocytes can be defined. Together, these platforms enable identification of the signaling features required to sustain thermogenic competence and underscore the need for technologies capable of reversible, spatially defined control of adipocyte signaling in diseased tissue. However, because iPSC-derived adipocyte models partially reset age- and disease-associated features and lack key aspects of the native adipose microenvironment, complementary validation in primary human cells, organoid or co-culture systems, and *in vivo* models will remain essential.

## Outlook: redefining success in thermogenic therapies

Looking forward, the challenge is not simply to identify additional thermogenic pathways, but to develop technologies capable of engaging these pathways with the spatial, temporal, and reversible control required to restore cellular competence. In this context, optogenetic approaches offer a strategy to directly engage thermogenic signaling within adipocytes with spatial and temporal precision that is difficult to achieve when non-cell-autonomous constraints are present. Consistent with principles from developmental biology, effective control of cell behavior depends not only on which signals are delivered, but on when, where, and for how long they are applied within a permissive cellular context. Emerging light-inducible and optogenetic approaches exemplify this shift by providing experimental tools to precisely modulate defined signaling pathways with controlled timing, amplitude, and anatomical restriction that cannot be achieved with systemic pharmacology. Conceptual frameworks have begun to articulate how optogenetic control can be leveraged to interrogate thermogenic adipocyte function with this level of precision (Brown, 2023), and experimental studies demonstrate that temporally defined optogenetic activation is sufficient to drive thermogenic programs in adipocytes without chronic stimulation (Doucette et al., 2023). More specifically, these approaches are best viewed as tools to test whether restoring specific aspects of cellular competence improves the durability of thermogenic responses, rather than as strategies that would broadly correct all impaired processes. Consistent with this experimental potential, independent *in vivo* studies using wireless optogenetic systems show that spatially restricted, non-canonical activation of adipose tissue can protect against obesity (Tajima et al., 2020). Coupled with renewable human cell platforms and increasingly sophisticated delivery technologies, these approaches provide a framework for testing whether restoring cellular competence can improve thermogenic responsiveness in disease-relevant models. In this view, success in thermogenic intervention strategies may ultimately be defined not only by acute increases in energy expenditure, but by recovery of the cellular plasticity required for durable thermogenic responsiveness. Accordingly, the conceptual model in Figure 1 depicts precision control as targeting discrete, testable components of cellular competence rather than implying simultaneous restoration of all impaired pathways. More broadly, this perspective reframes thermogenic failure as a problem of cellular competence and highlights the need for experimental strategies that restore the adaptive capacity of adipocytes in metabolic disease.

## Data Availability

The original contributions presented in the study are included in the article/supplementary material, further inquiries can be directed to the corresponding author.
